# Morbid Anatomy

**Published:** 1833-10-01

**Authors:** 


### III. *(bis.)*

### WORKS ON MORBID ANATOMY.

I. Anatomie Pathologique du Corps Humain. Par *J. Cruveilhier*. Dix-septieme livraison, &c.

II. Principles and Illustrations of Morbid Anatomy. By *J. Hope*, M.D. F.R.S. &c. &c. Part VI.

These have been published since our last number, and it is not a little singular, that at one and the same time, fasciculi of three distinct works on morbid anatomy should appear periodically in London and in Paris. We have alluded to the characteristic features of each, and we need not do more at present than give a succinct account of the fasciculi before us.*

---

* We had intended to have noticed some valuable French works which we have received, in the present number. Circumstances have compelled us to defer them until our next, when the recent volumes of M. Velpeau, M. Dugés, and the Memoirs of the Academy of Medicine will be brought before our readers.

And first of that of M. Cruveilhier. The subjects illustrated are—Diseases of the Brain in the Fœtus—Diseases of the Bladder and Prostate—Diseases of the Muscles—Diseases of the Heart and Aorta—and Diaphragmatic Hernia. These subjects are for the most part treated so briefly, and are of so miscellaneous a character, that we have thought it better to notice them in another part of this Journal, to which we must refer our readers.* The one which we shall select for notice here is,—

### Puerperal Inflammation of the Muscles and Synovial Membranes.

The researches of M. Dance, M. Velpeau, and others on the Continent, and of Dr. Lee in this country, have done much to disseminate accurate notions of what is termed the puerperal fever. How inexact and unphilosophical those notions were, prior to the recent dissections, might readily be proved by a reference to the over-rated work of the late Dr. Gooch. The secondary inflammations after parturition resemble in almost every respect the inflammatory deposites that succeed to injuries and operations. It is well known that after their occurrence or performance, the lungs, the liver, the serous and synovial cavities, the cellular tissue, and the muscles, especially the gastrocnemii, are the seats of inflammation, sloughing, or collections of matter.

M. Cruveilhier terms this affection of the muscles or of the synovial membrane, after parturition, rheumatic. The analogy is evidently false, and the term reprehensible. With this protest, we will, for convenience sake, employ his term " puerperal rheumatism."

Puerperal rheumatism appears at different periods after accouchement—sometimes immediately—sometimes before, during, or after the milk fever. It may occur alone—in the course of acute or chronic puerperal peritonitis—or of acute or chronic puerperal pleurisy—or during the convalescence from these diseases. It often coexists with the presence of pus in the lymphatic uterine vessels.

Being often attended with œdema, it is frequently confounded with phlegmatia dolens, which appears to M. Cruveilhier to be always the effect of phlebitis.

Puerperal rheumatism has a marked deposition to terminate in the formation of pus, especially in hospitals. The purulent collections multiply, and when one seems relieved, others appear, the patient dies, and dissection discloses the muscles infiltrated with pus, the articular and thecal synovial membranes filled with it, and the articular cartilages ulcerated and destroyed.

The puerperal inflammation of the synovial membranes may be latent, like that of the pleura and peritoneum.

Puerperal rheumatism may exclusively attack the cellular membrane enveloping, and forming a sort of atmosphere to the muscles.

The phlegmatia dolens, or œdema lactantium, is not a primitive disease, but merely a symptom, either of phlebitis, or of inflammation beneath the fascia.

---

* Periscope, p. 512, et seq.

M. Cruveilhier is tempted, from many facts, to believe that synovial inflammations are often a sequence to formation of pus in the uterine lymphatics and their glands.

A fact related by M. Cruveilhier appears to him to prove—1, that inflammation and purulent effusion in the uterine lymphatics may be cured; 2, that pus may exist with absolute impunity in these vessels, or, at all events, with greater impunity than in the veins; 3, that inflammation and purulent collection in the lymphatics may occur, independently of peritonitis.

Three cases are related by M. Cruveilhier, and delineations of the morbid appearances in the muscles are appended. We will cast a rapid glance at the cases.

*Case* 1. The patient aged 31. Accouchement Sept. 4, 1830. On the next day shivering, followed by slight abdominal pain. On the 6th, slight hypogastric pain; pulse 120. On the 7th, pain in the left leg, increased on pressure—thirst—abdomen tympanitic. On the 8th, pain in the left foot. In the night of the 9th, delirium. On the 10th, expression rather altered, tongue dry, some involuntary stools, pulse 100. 11th. Patient thinks herself getting well—slight deririum. 12th. Vomiting and pain at the epigastrium. 13th. State of prostration. Death in the evening, 9th day after confinement.

*Autopsy.* Slight effusion of lymph and pus in the cavity of the peritoneum—pus in that of the pelvis. On the sides of the uterus, in the broad ligaments, and along the fallopian tubes, lymphatics, filled with pus. Uterine veins healthy. Much blackish fluid in the left side of the chest. Subcutaneous cellular tissue of the left leg infiltrated with yellowish serum. Articulations of the ankle and tarsal bones filled with pus. Tendinous sheaths of the flexor communis, flexor proprius, and tibialis posticus filled with pus.

*Case* 2. Patient aged 25. Second accouchement March 3d, 1832. No complaint till the evening of March 11th, when there were pain and swelling of the left knee. 12th. These symptoms increased, with fever, pulse small and frequent, pulmonary catarrh. 14th. Œdema, with pain, of the whole lower extremity—face sallow—respiration hurried. 20th. Œdema less, knee and whole lower limb, especially the leg, tender on pressure—pulse frequent, sometimes intermitting. 22d. More violent pain in the knee—small phlyctenæ on the back of the thigh and leg—sore throat. During the next days, sloughing, where the phlyctenæ were. On the 30th, the knee-joint opened with caustic, and much pus let out. Death in the night of the 31st.

*Autopsy.* Large collection of pus between the muscles of the thigh and the bone, communicating with the interior of the knee-joint, and extending down the leg, above and beneath the soleus. Pneumonia at the base of the right lung. Pus in the ovaria. Pus in the lymphatics of the broad ligaments—in the lumbar glands—in the lymphatics on the psoas—in the glands at the obturator foramen.

*Case* 3. A washerwoman, aged 25. Third accouchement Jan. 19th, 1832. In a few hours afterwards acute hypogastric pain. Next day tenderness on pressure of the hypogastric and iliac region—pulse frequent. 21st. Pain of abdomen, though not constant—abdomen swollen—herpes labialis—pulse very frequent. 22d. Slight delirium. 25th. Pain in the wrist joint, with little tumefaction—agitation. In the following days the swelling increased and extended to the hand and forearm. Feb. 5th. Fluctuation around the joint. 10th. Opening made, and great quantity of pus let out. Pain in the inferior part of the leg and foot. 14th. Two subcutaneous abscesses on the inside and outside of the leg—another in the substance of the muscles on the posterior part of the forearm. Pyrexia and sweats. 18th. Death in great anguish.

*Autopsy.* Pus in the radio-carpal and carpal articulations, along the tendons and muscles of the fore-arm, in the substance of the muscles of the thumb and little finger. Cartilages of the articulations absorbed. Abscess in the extensor communis digitorum. Abscesses in the subcutaneous cellular tissue of the leg. Pus in a dilated lymphatic on the right edge of the uterus.

We have not related the treatment of these cases, because they are intended to illustrate pathological facts—the lesions that may follow parturition, and the general character of the symptoms that attend them. It is almost unnecessary for us to repeat, that they are, to all appearance, identical with the secondary lesions from injuries and from operations.

We now proceed to the consideration of the second work upon our list, that of our able friend Dr. Hope.

It is the sixth faciculus of Dr. Hope's work, published in August of the present year. It is dedicated to diseases of the alimentary canal below the diaphragm. These diseases are divided into, 1. Lesions of circulation. 2. Lesions of nutrition. 3. Lesions of secretion. In the first division are comprised hyperæmia and anæmia; in the second, hypertrophy, atrophy, softening, ulceration, perforation, and changes of capacity; in the third, morbid secretions in the mucous membrane, and morbid secretions beneath the mucous membrane. This will give an idea of the subjects comprised in this fasciculus. We will endeavour to select such portions as may appear particularly curious or important.

*Hyperæmia, or Redness of the Alimentary Canal.*

It is highly necessary to determine what is, and what is not, a natural degree of redness. Those who are in the habit of witnessing post-mortem examinations, must often have had occasion to remark the differences of opinion on this point. Dr. Hope very properly draws attention to what may be considered the natural state of the intestinal mucous membrane, in life and death.

"The intestinal mucous membrane of a living animal, during a tranquil state of the circulation, is observed to be of a red tint somewhat deeper than that of the mucous membrane of the cheek in a healthy man. This tint is replaced by uniform paleness, or, at the utmost, by a delicate rosy tinge when the animal is deprived of life without much loss of blood, which causes preternatural paleness, and without asphyxia, which causes mechanical injection. It thus appears that the mucous membrane, like the skin, tends to become pale after death. Accordingly, in the human species, as well as in animals, it has been found of this colour in most cases of accidental death occurring during a state of perfect health.

The pale colour, however, has not the same shades in all parts of the canal and at all ages. In the stomach, and still more in the great intestine, of the adult, it presents a dead white hue, while in the duodenum and jejunum it is of an ashy or greyish white, which diminishes towards the end of the ileum. Such, then is the natural colour in the adult.

In the fœtus and very young infant, the membrane is tinged of a rose-colour, which, gradually diminishing, is replaced in children by a milky and satiny whiteness; this becomes dimmer towards puberty, and, in the adult, passes into the ashy-grey shade above described. In the aged, the grey colour becomes more decided and general, being in some measure dependent on the dilated and congested state of the submucous veins, which impart a colour to the super-imposed membrane. In extreme old age, however, and in young children who have died of marasmus, the *maximum* degree of paleness is sometimes observed, being connected with the anæmia which exists under both these circumstances. I recently saw the intestine of an old man as white as if it had been long in maceration." 148.

Such, according to Dr. Hope, and we suppose that it may be considered a fair approximation to truth, is the natural tint of the mucous membrane. The next point considered by our able and industrious author is hyperæmia, independent of disease in the canal. This includes, of course, two conditions, which have attracted much attention and excited some discussion of late years—staining and congestion.

The causes enumerated by Dr. Hope, as capable of imparting a red colour to the alimentary canal, and operating before death, are—

1. *Digestion.* The redness which this process is well known to occasion continues, to a certain extent, after death, for, in the part where the process was going on at the moment of dissolution, the mucous membrane presents a beautifully delicate rose colour. This is not very appreciable below the duodenum.

2. All circumstances opposing the free return of the venous blood from the intestines to the right side of the heart; namely, asphyxia,—obstructions of the vena portæ, whether by disease of the liver, tumours situated in the course of its principal intestinal divisions, or coagula in the vein itself; strangulation of the intestines; organic disease of the heart, and affections of the lungs, attended with considerable dyspnœa. Asphyxia operates more or less in almost every case of slow and difficult dissolution. The redness from these causes is less, of course, in proportion as the body is exsanguined. The degree varies according to the intensity of the cause. In the lowest degree there is nothing more than a bluish intumescence of the larger submucous veins; certain branches in the mucous membrane next become injected, presenting the different gradations of ramiform vascularity; finally, the capillary net-work is completely affected, and the result is a uniform red stain. These characters generally pervade a large extent of intestine: but if circumscribed, which is rare, they may form bands, patches, streaks, spots, or mere points: every appearance, in short, which we see produced by inflammation.

Redness, occasioned after death, is referable to two principal causes—1, gravitation of the blood; 2, its transudation through the parietes of its vessels.

Gravitation to the most dependent parts of the intestines invariably takes place, in whatever position the animal may have been placed. It commences immediately after death, and continues till the blood has cooled down to the point of coagulation, and, consequently, will be greater where the temperature has been artificially or naturally maintained longer than usual, or where the blood remains preternaturally fluid. It will also be greater in subjects abounding in blood, than in those affected, previous to death, with congestion of the portal system.

Transudation of blood through the walls of the vessel commences simultaneously with putrefaction, and takes place in vessels of all calibre. Thus it forms the red spots almost always observed along the veins of the greater extremity of the stomach, when the subject is examined more than thirty-six or forty hours after death. These spots, extending and coalescing, constitute multiform groups, bands, streaks, &c.; and eventually, not only the tract of the vessels, but the whole surface of the organ becomes more or less uniformly tinged. When there is much congestion of the vessels, the mucous membrane, and, equally, all the other coats may become thoroughly soaked and reddened. Complete transudation may take place within the brief space of twenty-four hours, provided putrefaction be favoured by a high summer temperature and atmospheric humidity. Under such circumstances blood may even transude into the interior of the canal. Transudation from the spleen also, particularly when the blood of this organ is very liquid, may communicate a stain to the stomach.

Our author appends from Billard a comparative table of the characters of congestive and inflammatory redness.

Blood, exposed to gases developed in the intestines, undergoes such changes of colour as it would do, if exposed to the same gases in a bladder. The prevailing colours are various tints of green and brown. A rich yellow colour is not unfrequently imparted to the stomach and intestines by the bile, which produces a stain of the mucous membrane, incapable of being removed by washing. Yellow patches or bands of greater or less extent are sometimes seen in the mucous membrane of the duodenum or jejunum in its healthy state. Billard thinks that they take place subsequent to death.

Dr. Hope proceeds to describe hyperæmia of the alimentary canal from disease. The alterations of colour produced by inflammation are reducible to four principal species, namely, red, brown, slate-coloured, and black, between each of which there are intermediate shades.

The red colour presents several varieties of aspect which may be characterised as—1, ramiform injection; 2, capilliform injection; 3, redness in patches; 4, diffused redness; 5, speckled redness; 6, streaked redness. The redness produced by inflammation differs in this great feature from that produced by congestion—the capillaries are injected, while the vessels of large diameter are devoid of colour.

Ramiform injection is the result of a slight irritation only, and is either the first trace of a mild incipient inflammation, or the vestige of a more severe one which is subsiding. Capilliform injection implies a greater degree than the former, the capillary ramifications being affected. Redness in patches is an evidence of inflammation of high intensity. Seated in the great intestines, particularly at their lower part, it is the most common morbid alteration produced by simple dysentery; in the ileum it is attended with symptoms of enteritis, and is common in fever. Passive redness in patches is rare. Diffusive redness is the result of a higher degree of inflammation than any other variety. It is completely removed by maceration for twenty-four hours, an evidence of the acuteness of the inflammatory action. This is more likely than the other varieties to be confounded with passive congestion, because the latter is likewise commonly diffuse. The mucous membrane may acquire a diffuse red stain from blood effused into the canal, and from certain colouring liquids taken either as food or medicine, for instance, black currants, infusion of logwood, &c.

Speckled redness has not inaptly been compared to the section of an inflamed brain. It is probably produced by extravasation from a certain number of minute vessels of the mucous membrane, taking place on the first inflammatory impulse. It may therefore be regarded as the vestige of only slight inflammation. Streaky redness is not very common. The summits of wrinkles in the stomach, and of the valvulæ conniventes in the parts below are its ordinary seats.

Dr. Hope next alludes to two sub-varieties of hyperæmia, namely, of the villi, and of the follicles. The account of either of these is taken from Andral, and we need not transcribe it.

Dr. Hope remarks that it is difficult to determine in all cases whether the colouring of the alimentary canal depends on acute, or on chronic inflammation or irritation; but it may be stated in general terms, *first*, that the red colour belongs principally to acute disease, though it is not uncommon in chronic, as, for instance, in protracted diarrhœa; *secondly*, that the brown, slate-coloured, and black belong almost exclusively to chronic disease, though they may also be occasioned by acute inflammation, especially that produced by certain poisons.

Our author next proceeds to the description of the brown, the slate colour, and the black. This, in nine cases out of ten, according to Billard, originates in inflammation, and indicates a chronic state of it. It usually presents either a diffuse or a streaky and marbled aspect. When the result of inflammation, it is commonly attended with thickening and facility of detaching the mucous membrane; when following very acute inflammation, the membrane is softened, almost to a pulp. The slate colour is also found to be in nine cases out of ten the result of chronic inflammation. Whether the black colour can result from stagnation alone, or whether the presence of some other morbid agent, as inflammation or irritation be necessary, cannot be positively determined.

"Of inflammatory hyperæmia, in general, it may be stated that certain parts of the alimentary canal are more subject to it than others. The stomach and the lower portion of the ileum come first in the scale of frequency; then follow, successively, the cæcum, the colon, the rectum, the duodenum, the superior part of the ileum, and the jejunum. (Andral.) This scale accords with the results of my own observation." 163.

The remainder of this Fasciculus is occupied with the consideration of softening of the alimentary canal. The following is the natural condition of the mucous membrane, according to Andral.

"In the stomach, says Andral, we may allow the mucous membrane to be of the natural thickness, when, on making an incision in it, without dividing the sub-mucous cellular tissue, we can easily detach pretty considerable shreds with a forceps: the shreds should be larger in the pyloric, than in the splenic portion.

In the duodenum, a difference in the nature of the membrane, probably connected with the greater abundance of mucous follicles, renders us unable to detach so considerable shreds as in the stomach.

In the rest of the intestines, the rectum excepted, the mucous membrane, even in its natural state, breaks and tears whenever we attempt to detach any portion of it. If the membrane should become attenuated in consequence of a general defect of the nutritive powers, it at the same time becomes softer, without the previous or present existence of any process of irritation." 164.

Softening from putrefaction, and softening by the gastric juice, are considered previously to softening by disease. The former seldom takes place before the eighth or tenth day, and therefore if softening is observed much earlier there has been some other cause. Softening from the gastric juice we need not particularly advert to, as the subject has long attracted attention.

Softening by disease may be confined to the mucous membrane alone, or may extend through all the coats.

Softening of the mucous membrane alone is more frequent and more marked in the stomach than elsewhere. Sometimes it is general, more frequently partial, and the splenic portion of the organ is most liable to it. The veins are often dilated, and the blood curdled in them. Occasionally this softening forms only isolated spots, half an inch or less in diameter, of irregularly circular form, and either redder or whiter than the surrounding membrane. Sometimes softening occurs in lines, streaks, or sinuous bands. The colour is thought by Dr. H. to depend on the previous tint of the membrane. Softening may be the result of either acute or chronic inflammation, or of a process not manifestly inflammatory. When the result of acute inflammation, the symptoms are those of acute gastritis or enteritis. Softening from chronic inflammation is attended with the symptoms of chronic inflammation. Softening, however, may occur without symptoms of inflammation at all, in the advanced stages of many chronic diseases, especially of the lungs.

"I have so frequently noticed the following train of symptoms in the extensive Institution to which I am attached, and which, independent of patients, contains several hundred old people, that I am induced to offer Andral's account in his own words: 'Softening of the mucous membrane of the stomach appears to me to be a common affection in old people whose digestion becomes disordered, their health having previously been very good. Their appetite first diminishes, they then lose it entirely, and, soon after, begin to feel the greatest dislike to all kinds of food. They experience a constant feeling of uneasiness and weight, rather than actual pain, in the region of the stomach; and their tongue, which is usually natural, or else more or less thickly coated, grows red and dry occasionally. This state may continue for several months; the pulse then becomes more frequent, a considerable emaciation takes place, the strength rapidly declines, and the patients die without showing symptoms of a serious affection of any organ up to the last moments. On opening the body, there is nothing found but a more or less considerable softening of the mucous membrane of the stomach, with or without injection of its tissue.'

In the remainder of the alimentary canal, precisely as in the stomach, chronic softening is often extremely ambiguous in its symptoms; as it sometimes occurs independent of pain or derangement of function in any marked degree; and, on the other hand, these symptoms are of common occurrence independent of softening. Instead, therefore, of outstripping the progress of science by premature attempts to define the connexion between the lesion and its symptoms, it is more prudent to confine ourselves to observation until we are in possession of more satisfactory data." 171.

Softening of all the coats of the gastro-intestinal parietes has been accurately described by Cruveilhier, who saw it prevail epidemically amongst the young children at Limoges. This softening may present any colour, and may take place at any period of life. It occurs most frequently in the splenic portion of the stomach, but has also been found in various parts of the small intestines, cæcum, and colon.

We have noticed the letter-press of this fasciculus so freely, that we have no space for commendation of the plates. We can only say that they equal their predecessors, and that is no mean praise.

